# What's in a name? From PCOS to polyendocrine metabolic ovarian syndrome: A metabolic reframing, promise, controversies, and challenges ahead

**DOI:** 10.1016/j.metop.2026.100479

**Published:** 2026-05-20

**Authors:** Maria Dalamaga

**Affiliations:** Department of Biological Chemistry, School of Medicine, National and Kapodistrian University of Athens, 75 Mikras Asias, 11527, Athens, Greece

**Keywords:** Cardiometabolic risk, Consensus, Metabolic dysfunction-associated steatotic liver disease, Nomenclature, Polycystic ovary syndrome, Polyendocrine metabolic ovarian syndrome

## Abstract

In medicine, nomenclature shapes diagnostic reasoning, clinical pathways, research priorities, and patients' understanding of their condition. Polycystic ovary syndrome (PCOS), the most common endocrine disorder in women of reproductive age, affecting one in eight women and more than 170 million individuals worldwide, has long carried a name that foregrounds ovarian morphology and implies pathological cysts. This framing has narrowed clinical attention, contributed to delayed diagnosis in up to 70% of affected individuals, and obscured the broader endocrine, metabolic, psychological, and cardiometabolic dimensions of the syndrome. A recent global consensus process led by the Global Name Change Consortium, involving 56 organizations, patients, clinicians, and researchers, has renamed the condition polyendocrine metabolic ovarian syndrome (PMOS). The new terminology recognizes interacting disturbances in androgen production, insulin signaling, neuroendocrine regulation, and ovarian function, while centering metabolic features such as insulin resistance, dysglycemia, dyslipidemia, cardiovascular risk, and metabolic dysfunction-associated steatotic liver disease. This editorial examines the historical evolution of diagnostic criteria from the 1990 NIH criteria through the 2003 Rotterdam consensus to the 2023 International Evidence-Based Guideline, reviews the structured renaming process, and draws parallels with the transition from non-alcoholic fatty liver disease (NAFLD) to metabolic dysfunction-associated steatotic liver disease (MASLD). It discusses controversies surrounding the new name, implementation challenges, and the infrastructure needed to ensure that PMOS improves diagnostic accuracy, broadens multidisciplinary care, and reduces stigma without compromising the existing evidence base.

In medicine, nomenclature is not merely descriptive. It shapes diagnostic reasoning, clinical pathways, research priorities, funding streams, medical education, and, importantly, patients’ understanding of their condition. When a name is biologically inaccurate, it may narrow clinical attention and reinforce misconceptions. The renaming of polycystic ovary syndrome (PCOS) as polyendocrine metabolic ovarian syndrome (PMOS) is therefore more than a semantic change [1]. It represents an evidence-based attempt to align terminology with contemporary understanding of a common multisystem disorder affecting one in eight women and more than 170 million women during their reproductive years worldwide [[1], [2], [3]].

## 1 The journey to renaming: A historical perspective

The evolution of PCOS diagnostic criteria reflects a gradual broadening of the syndrome from a primarily reproductive construct to a more complex endocrine-metabolic disorder. The 1990 National Institutes of Health criteria emphasized clinical or biochemical hyperandrogenism together with oligo-amenorrhea, after exclusion of related disorders [4]. The 2003 Rotterdam criteria expanded the diagnostic framework by allowing diagnosis when two of three features were present: hyperandrogenism, ovulatory dysfunction, or polycystic ovarian morphology on ultrasound [4]. The 2023 International Evidence-Based Guideline further refined diagnosis by incorporating anti-Müllerian hormone (AMH) as an alternative to ultrasound for defining polycystic ovarian morphology in adults, while clarifying that in adolescents, both hyperandrogenism and ovulatory dysfunction are required, with ultrasound and AMH not recommended because of poor specificity [5,6].

Despite these diagnostic advances, the name remained anchored to ovarian morphology. This was increasingly discordant with the biology of the condition. The term “polycystic ovaries” is problematic because the ultrasound appearance reflects an excess of small arrested follicles rather than true pathological ovarian cysts. Moreover, polycystic ovarian morphology is neither required nor sufficiently specific for diagnosis. By foregrounding the ovary and “cysts,” the previous name reinforced a narrow gynecological interpretation and obscured the broader endocrine, metabolic, psychological, dermatological, and cardiometabolic dimensions of the syndrome [1,2].

## 2 What happened: A global consensus process

The Global Name Change Consortium describes a structured global consensus process designed to move the condition beyond the narrow implications of “polycystic ovary.” The initiative involved 56 academic, clinical, and patient organizations, with participation from people with PCOS, multidisciplinary health professionals, and researchers across world regions. The process included global surveys, modified Delphi methods, nominal group technique workshops, marketing and communication analyses, and implementation planning. The transition to PMOS is intended to occur over approximately 3 years, with integration into the 2028 update of the International Evidence-Based Guideline [1].

The rationale for renaming had been building for more than a decade, including the 2012 National Institutes of Health recommendation that the name should better reflect the condition's biology [1,2]. In the global consensus process, the approach prioritized on survey was adoption of a new, symptom-based name, favored by 86% of people with PCOS and 71% of health professionals [1]. The terms “endocrine” or “polyendocrine” and “metabolic” received broad support, reflecting recognition that the syndrome extends well beyond ovarian morphology [1,2].

This support was not merely semantic. The previous name has contributed to misunderstanding among patients, clinicians, policy makers, and funders. Up to 70% of affected individuals remain undiagnosed, and the ovarian and reproductive framing has contributed to poorly integrated care, delayed diagnosis, stigma, and misalignment of research and policy priorities. Insulin resistance, androgen excess, dysglycemia, hypertension, dyslipidemia, cardiovascular risk, sleep apnea, and metabolic dysfunction-associated steatotic liver disease (MASLD) are central to the syndrome, yet these features were largely invisible in the name PCOS [1,2].

## 3 The new definition: A more accurate terminology

The new terminology more accurately reflects the biological architecture of the syndrome. “Polyendocrine” recognizes that PMOS involves interacting abnormalities in androgen production, insulin signaling, neuroendocrine regulation, AMH, and ovarian function. “Metabolic” appropriately brings insulin resistance, adiposity-related biology, dysglycemia, type 2 diabetes, hypertension, dyslipidemia, obstructive sleep apnea, cardiovascular risk, and metabolic dysfunction-associated steatotic liver disease into the central disease framework rather than treating them as secondary considerations [7]. “Ovarian” preserves the importance of ovulatory and follicular dysfunction without implying the presence of true ovarian cysts. “Syndrome” remains appropriate because the condition comprises a heterogeneous constellation of reproductive, metabolic, endocrine, dermatological, and psychological features [1,[8], [9], [10], [11]].

Importantly, the name change does not imply an immediate change in diagnostic criteria. Current guideline-based diagnosis still rests, after exclusion of conditions with overlapping features, on at least two of three adult criteria: hyperandrogenism, ovulatory dysfunction, and polycystic ovarian morphology on ultrasound or elevated AMH [5,12]. In adolescents, both hyperandrogenism and ovulatory dysfunction are required, with ultrasound and AMH not recommended until at least 8 years post-menarche [[13], [14], [15]].

## 4 Why this matters at the bedside

The clinical importance of this renaming becomes evident at the bedside. An adolescent with severe acne, irregular menstrual cycles, and biochemical hyperandrogenism should not be reassured that PMOS is excluded solely because ultrasound does not show “cysts.” Similarly, an adult treated for hirsutism or menstrual irregularity should not leave the clinic without assessment of metabolic risk, including glycemia, serum lipids, blood pressure, sleep apnea symptoms, endometrial protection, fertility goals, mental health, and the impact of weight stigma. The term PMOS may therefore help clinicians adopt a broader clinical lens and may help patients understand that their symptoms arise from an integrated endocrine-metabolic syndrome rather than from unrelated problems.

The cardiometabolic burden is substantial. Impaired glucose tolerance affects 30–35% of women with classic PCOS, and type 2 diabetes affects 8–10% [8]. Women with PCOS present lower HDL cholesterol and higher triglyceride and LDL cholesterol levels than women without the syndrome, differences that are at least partly independent of BMI [6]. The risk of endometrial cancer is estimated to be 2.7 times higher, with lifetime risk reported as high as 9% [8]. Subclinical vascular abnormalities, including impaired endothelial function and increased carotid intima-media thickness, have also been documented [8,9]. These established risks underscore the Consortium's framing of PMOS as a multisystem disorder in which metabolic and cardiovascular features are central, not peripheral [1].

## 5 Parallels and lessons: the NAFLD to MASLD transition

Metabolic medicine has recently experienced a comparable nomenclature transition in liver disease. Non-alcoholic fatty liver disease (NAFLD) was defined by exclusion and carried terms, namely “fatty” and “alcoholic”, that were often imprecise and potentially stigmatizing. Metabolic dysfunction-associated fatty liver disease (MAFLD) represented an effort to move toward positive metabolic criteria [[16], [17], [18], [19]]. More recently, multisociety consensus adopted metabolic dysfunction-associated steatotic liver disease (MASLD), defined by hepatic steatosis together with at least one cardiometabolic risk factor; metabolic dysfunction-associated steatohepatitis (MASH) replaced Nonalcoholic Steatohepatitis (NASH), and Metabolic dysfunction and alcohol-associated liver disease (MetALD) was introduced to describe the overlap between metabolic dysfunction and higher alcohol intake [[20], [21], [22]].

The liver nomenclature experience is instructive for PMOS. It showed how a biologically aligned name may reduce stigma and center metabolic risk, but also how terminology changes create predictable challenges: acronym fatigue, coding transitions, search-term discontinuity, uncertainty when comparing historical and contemporary cohorts, and debate about whether old and new labels classify the same individuals [16,20,23]. For example, a patient with steatosis, diabetes, and modest alcohol intake may be classified as having MASLD; with higher alcohol intake, MetALD; and with viral hepatitis and obesity, dual etiology must not be obscured by a metabolic label. Reassuringly, studies showed that virtually all individuals previously diagnosed with NAFLD met criteria for MASLD, minimizing diagnostic disruption, a precedent that may ease the PCOS-to-PMOS transition [23].

## 6 Controversies and challenges

Renaming is not without risk. PMOS is more biologically accurate than PCOS, but it is also longer, more technical, and initially unfamiliar. Some may question whether the term “ovarian” remains sufficiently inclusive for transgender and gender-diverse individuals, or for those beyond reproductive age. Others may worry that emphasizing metabolism could unintentionally intensify weight stigma, particularly if lifestyle counseling is delivered in a reductive or blame-based manner. The Global Name Change Consortium acknowledged these implementation challenges, including the need for culturally appropriate language, careful translation, and broader representation from underrepresented regions [1].

Paradoxically, the metabolic framing may actually protect a frequently overlooked subgroup. Approximately 20–50% of affected women are not obese, yet those with so-called 'lean PCOS' demonstrate insulin resistance, adverse adipokine profiles, and dyslipidemia compared to BMI-matched controls [24,25] A recent study found that nonobese and obese PCOS women had similar degrees of insulin resistance when assessed by the disposition index, suggesting the same underlying metabolic disorder regardless of body habitus [26]. These women are particularly vulnerable to diagnostic delay because clinicians may not suspect the syndrome in the absence of obesity, a misconception the old name reinforced. By centering endocrine and metabolic dysfunction rather than body weight, PMOS may help ensure that lean individuals receive appropriate metabolic screening rather than being dismissed as unlikely to have the condition.

Not all experts have historically supported a single unifying name. Some have argued for a “two-state solution,” retaining PCOS for predominantly reproductive phenotypes while assigning a different name to metabolically driven phenotypes [27]. Others have raised concerns about diagnostic confusion, implementation costs, and the risk that a new name could complicate literature searches, coding systems, and patient advocacy efforts. The Rotterdam criteria created four diagnostic phenotypes with differing reproductive and metabolic risk profiles, and some proposed alternatives, including “Syndrome XX,” “Female Metabolic Syndrome,” or “Metabolic Reproductive Syndrome”, were intended to better capture specific high-risk subgroups [27,28]. However, the consensus process prioritized a unified name that captures the multisystem biology of the condition while acknowledging that future work on phenotyping, clustering, genetic risk, and metabolic stratification remains essential [1,2].

## 7 Problems, perspectives, and solutions

The lesson from both the PMOS and MASLD transitions is clear: names must be accompanied by navigational tools. For several years, publications, electronic health records, trial registries, systematic reviews, and clinical guidelines should use transitional terminology such as “PMOS, formerly PCOS.” Search strategies must deliberately include both terms to preserve continuity with the existing evidence base. Disease classification systems, including International Classification of Diseases (ICD) and Systematized Nomenclature of Medicine (SNOMED), as well as medical curricula, patient-facing resources, grant-review panels, journal keywords, and advocacy materials, will require coordinated crosswalks. Without this infrastructure, a name intended to improve recognition could inadvertently affect research, clinical documentation, and patient communication.

The consensus process anticipated many of these risks. Its implementation strategy includes publication and academic dissemination; development of multilingual resources for people with PCOS and clinicians; coordinated global communication and engagement; integration into electronic health records and health-care education systems; alignment with policy agencies, research funders, and journal processes; and formal engagement with international classification bodies, including World Health Organization (WHO), for adoption in the ICD [1,2]. Patient advocacy organizations will need to rebrand while maintaining continuity for individuals who have long identified with PCOS [1]. Researchers will need to protect searchability and cohort comparability during the transition. The 3-year implementation period leading into the 2028 International Guideline update offers a pragmatic roadmap for staged adoption rather than abrupt replacement [1].

## 8 The promise of PMOS

The promise of PMOS is substantial. The new name invites endocrinologists, gynecologists, reproductive specialists, dermatologists, primary care physicians, hepatologists, cardiologists, psychologists, dietitians, and other clinicians to recognize a shared multisystem condition rather than a disorder confined to the ovary. It also reminds metabolism researchers that PMOS is not a niche reproductive diagnosis, but a lifelong endocrine-metabolic syndrome with reproductive, dermatological, psychological, and cardiometabolic manifestations.

By reframing the condition beyond reproductive health, the new terminology may broaden research priorities, improve medical education across specialties, strengthen metabolic and cardiovascular screening, and reduce the confusion and stigma experienced by many affected individuals.

## 9 Looking forward

So, what is in a name? Sometimes a diagnosis. Sometimes a referral. Sometimes a glucose tolerance test, a serum lipid panel, a research grant, or relief from blame. PMOS will not, by itself, improve outcomes. However, a more accurate name may change the questions clinicians ask, the risks they screen for, the clinics they build, and the respect with which they listen.

The renaming of PCOS to polyendocrine metabolic ovarian syndrome represents an important moment in women's metabolic health. It acknowledges that nomenclature shapes research priorities, funding allocation, clinical recognition, and patient experience. As with the transition from NAFLD to MASLD, the success of PMOS will depend not only on the accuracy of the new term, but also on careful implementation, diagnostic continuity, education, and sustained engagement with patients and clinicians. The task now is to ensure that PMOS does not become merely a new acronym, but a more accurate clinical framework. If implemented thoughtfully, it can help shift care from episodic symptom management toward lifelong, multidisciplinary prevention and treatment. For a condition that has long been minimized, misunderstood, and fragmented across specialties, that change is not cosmetic. It is clinical.

## Funding

This work did not receive any specific grant from funding agencies in the public, commercial or not-for-profit sectors.

## Conflicts of interest

No conflict of interest to declare.
